# Ten-year results of an international external quality control programme for measurement of anti-tuberculosis drug concentrations

**DOI:** 10.1093/jac/dkae105

**Published:** 2024-04-06

**Authors:** Ralf Stemkens, Chaima Mouhdad, Eric J F Franssen, Daniel Touw, Jan-Willem Alffenaar, Lindsey H M Te Brake, Marieke G G Sturkenboom, Rob E Aarnoutse

**Affiliations:** Department of Pharmacy, Research Institute for Medical Innovation, Radboud University Medical Center, Nijmegen, The Netherlands; Department of Pharmacy, Research Institute for Medical Innovation, Radboud University Medical Center, Nijmegen, The Netherlands; Department of Clinical Pharmacy, OLVG Hospital, 1066 CX Amsterdam, The Netherlands; Drug Analysis and Toxicology section (KKGT), Dutch Foundation for Quality Assessment in Medical Laboratories (SKML), Nijmegen, The Netherlands; Department of Clinical Pharmacy and Pharmacology, University Medical Center Groningen, Groningen, The Netherlands; Department of Clinical Pharmacy and Pharmacology, University Medical Center Groningen, Groningen, The Netherlands; Faculty of Medicine and Health, School of Pharmacy, The University of Sydney, Sydney, NSW, Australia; The University of Sydney Institute for Infectious Diseases, Sydney, NSW, Australia; Department of Pharmacy, Westmead Hospital, Sydney, NSW, Australia; Department of Pharmacy, Research Institute for Medical Innovation, Radboud University Medical Center, Nijmegen, The Netherlands; Department of Clinical Pharmacy and Pharmacology, University Medical Center Groningen, Groningen, The Netherlands; Drug Analysis and Toxicology section (KKGT), Dutch Foundation for Quality Assessment in Medical Laboratories (SKML), Nijmegen, The Netherlands; Department of Pharmacy, Research Institute for Medical Innovation, Radboud University Medical Center, Nijmegen, The Netherlands; Drug Analysis and Toxicology section (KKGT), Dutch Foundation for Quality Assessment in Medical Laboratories (SKML), Nijmegen, The Netherlands

## Abstract

**Objectives:**

Participation in an external (interlaboratory) quality control (QC) programme is an essential part of quality assurance as it provides laboratories with valuable insights into their analytical performance. We describe the 10 year results of an international QC programme for the measurement of anti-tuberculosis (TB) drugs.

**Methods:**

Each year, two rounds were organized in which serum (or plasma) samples, spiked with known concentrations of anti-TB drugs, were provided to participating laboratories for analysis. Reported measurements within 80%–120% of weighed-in concentrations were considered accurate. Mixed model linear regression was performed to assess the effect of the measured drug, concentration level, analytical technique and performing laboratory on the absolute inaccuracy.

**Results:**

By 2022, 31 laboratories had participated in the QC programme and 13 anti-TB drugs and metabolites were included. In total 1407 measurements were reported. First-line TB drugs (isoniazid, rifampicin, pyrazinamide and ethambutol) represented 58% of all measurements. Overall, 83.2% of 1407 measurements were accurate, and the median absolute inaccuracy was 7.3% (IQR, 3.3%–15.1%). The absolute inaccuracy was related to the measured anti-TB drug and to the performing laboratory, but not to the concentration level or to the analytical technique used. The median absolute inaccuracies of rifampicin and isoniazid were relatively high (10.2% and 10.9%, respectively).

**Conclusions:**

The 10 year results of this external QC programme illustrate the need for continuous external QC for the measurement of anti-TB drugs for research and patient care purposes, because one in six measurements was inaccurate. Participation in the programme alerts laboratories to previously undetected analytical problems.

## Introduction

TB remains a major global health problem, with 10.6 million new cases and 1.3 million deaths worldwide in 2022.^1^ Adequate anti-TB drug concentrations in plasma or serum are crucial because they are the intermediary link between administered drug doses and the eventual drug effects of anti-TB drugs. Indeed, inadequate exposure to anti-TB drugs is a risk factor for suboptimal response and the development of resistance, whereas unduly high concentrations may cause adverse effects.^2^

For this reason studies evaluating the pharmacokinetics and concentration–effect relationships of anti-TB drugs, as well as drug interactions and/or food–drug interactions of these drugs, are essential during development of new anti-TB drugs and for dose optimization of existing drugs.

Achieving adequate anti-TB drug concentrations is also relevant in actual TB treatment. Therapeutic drug monitoring (TDM), i.e. dose individualization based on measurement and interpretation of drug concentrations, is performed in selected TB referral centres around the world. Studies describing the benefits of TDM in clinical practice have accumulated over the years,^3,4^ its availability across the world is spreading,^5^ and the application of TDM has been incorporated in clinical standards for TB treatment and in guidelines.^6^

Clearly analytical methods to measure anti-TB drugs are required for both pharmacokinetic studies and TDM. Commercial (semi-)automatic immunoassays are not available for the measurement of anti-TB drugs. As a result, laboratories need to develop and internally validate their own methods. Participation in an external (interlaboratory) quality control (QC) or proficiency testing programme is an essential part of quality assurance, as it provides laboratories with valuable insights into their analytical performance.^7^ It is also a requirement for medical laboratories.^8^ In 2012, the first global external QC programme for measurement of anti-TB drugs was initiated by the Drug Analysis and Toxicology section (KKGT), a part of the Dutch Foundation for Quality Assessment in Clinical Laboratories (SKML).^7^

The aim of this study was to describe the first 10 years of this programme including an assessment of the performance of participating laboratories as well as an evaluation of factors that may contribute to the inaccuracy of anti-TB drug measurements.

## Methods

### Description of the QC programme

The design of the QC programme was described previously.^7^ Each year, two rounds were organized in which serum (or plasma) samples, spiked with known concentrations of anti-TB drugs, were provided to participating laboratories for analysis (only one round was organized in the first 2 years). Initially, the programme included six anti-TB drugs: isoniazid, rifampicin, pyrazinamide, ethambutol, linezolid and moxifloxacin. Since the introduction of the programme several drugs (levofloxacin, rifabutin, bedaquiline and clofazimine) and metabolites (acetyl-isoniazid, desacetyl-rifampicin and desacetyl-rifabutin) have been added.

All drug substances were of analytical quality with a high purity as specified in the certificate of analysis (>95%), which was corrected for during the preparation of stock solutions. Anti-TB drugs were weighed using calibrated analytical balances and dissolved in organic fluid/water using calibrated pipettes and volumetric flasks. Blank serum (first two rounds) or plasma from healthy volunteers was obtained from the Dutch Blood Bank (Sanquin, The Netherlands). QC samples were prepared by spiking blank serum or plasma with stock solutions of anti-TB drugs at concentrations ranging from the low to the high therapeutic or toxic range of the drug. Due to the instability of isoniazid in plasma, QC samples for this drug were prepared in water since 2019. Participants received instructions to mix this isoniazid-containing QC sample with blank plasma before analysis. All QC samples were dispensed in polypropylene tubes. After preparation, QC samples were freeze-dried (first two rounds only) or immediately frozen at −80°C until shipment. Stability of samples under these conditions had been assessed before (R. Aarnoutse and L. te Brake, unpublished data). Before shipment to the participants, the samples were analysed with validated analytical methods and approved for release to the programme if the deviation of the measured concentrations was ≤10% of the weighed-in concentrations. Apart from the first freeze-dried samples, all materials were shipped on dry ice to participating laboratories because of the instability of some of the drugs.

The samples of each round were accompanied by one or two clinical cases that served educational purposes. The mock cases reflected real-life TDM practice of patients with drug-susceptible or MDR-TB. Participants were asked to provide dosing advice via a multiple-choice question based on concentrations of anti-TB drugs measured in the received samples. Participants had to submit their analytical results and answers to the clinical cases before the annual deadlines. Assessment of turnaround times was not part of the QC programme. Participants received feedback within 6 weeks, which included an evaluation of the case by the programme coordinator.

### Statistical analyses

During the programme, all laboratory measurements were standardized to percentages relative to the weighed-in concentrations, which were considered true values, by the following formula: (measured concentration/weighed-in concentration) × 100%. Measurements within 80%–120% of the true concentrations were defined as accurate, based on guidelines for bioanalytical method validation and maximal allowable error specifications for the lowest level of quantification.^9,10^ This range is also commonly applied by external QC programmes from the KKGT section of the SKML. The percentage inaccuracy from the true concentrations was calculated by subtracting 100% from these percentages.

Descriptive statistics were performed to assess the percentage of accurate measurements and the median absolute inaccuracy by (i) drug, (ii) concentration level, (iii) analytical technique and (iv) performing laboratory. As to the concentration level, the weighed-in concentrations were divided in tertiles for each drug and classified as low, medium or high. This division was deemed suitable for drugs that were included in at least nine rounds (i.e. at least three weighed-in concentrations were available for each category). Mixed model linear regression was performed to test the effect of the four above-mentioned factors on the absolute inaccuracy. The drug, concentration level and analytical technique were included as fixed factors and the performing laboratory as random factor. All statistical analyses were performed using SPSS version 27.0 (SPSS Inc., Chicago, IL, USA).

## Results

### Participating laboratories and overall results

In the 10 year period from 2012 to 2022 (in 2013 no rounds were organized) a total of 31 laboratories, representing 18 countries and five continents (Figure S1, available as Supplementary data at *JAC* Online), participated in at least one round of the programme, with a median of 13 participants per year (range 7–18). During this period 18 rounds were organized and a total of 1407 measurements were collected. Most measurements were collected for the first-line anti-TB drugs rifampicin, isoniazid, pyrazinamide and ethambutol (combined, *n* = 817; 58%), followed by linezolid (*n* = 155; 11%) and moxifloxacin (*n* = 149; 10.6%) (Table 1). Other compounds each represented less than 10% of all measurements. LC-MS was the most commonly used analytical technique (68.2%), followed by HPLC with other modes of detection (24.0%) and GC-MS (1.6%). The analytical technique was not reported for 87 measurements (6.2%, Table 1).

**Table 1.** Overall performance of the QC programme for measurement of anti-TB drugs

		Analytical method^a^			Performance			
Drug	Analyses, *N* (% of total)	LC-MS, *n* (%)	HPLC, *n* (%)	GC-MS, *n* (%)	Median absolute inaccuracy, % (IQR %)	Accurate, *n* (%)	<80%, *n* (%)	>120%, *n* (%)
*First-line drugs and their metabolites*								
Rifampicin	223 (15.8)	148 (66.4)	63 (28.3)	NA	10.2 (4.2–20.1)	168 (75.3)	29 (13.0)	26 (11.7)
Desacetyl-rifampicin	18 (1.3)	13 (72.2)	5 (27.8)	NA	20.8 (11.1–48.0)	11 (61.1)	3 (16.7)	4 (22.2)
Rifabutin	64 (4.5)	50 (78.1)	14 (21.9)	NA	8.4 (4.4–14.7)	53 (82.8)	6 (9.4)	5 (7.8)
Desacetyl-rifabutin	13 (0.9)	13 (100)	NA	NA	6.3 (3.6–17.6)	11 (84.6)	1 (7.7)	1 (7.7)
Isoniazid	209 (14.9)	134 (64.1)	61 (29.2)	NA	10.9 (4.9–21.5)	152 (72.7)	29 (13.9)	28 (13.4)
Acetyl-isoniazid	35 (2.5)	33 (94.3)	2 (5.7)	NA	6.2 (3.9–9.5)	32 (91.4)	2 (5.7)	1 (2.9)
Pyrazinamide	196 (13.9)	138 (70.4)	46 (23.5)	2 (1.0)	7.0 (3.2–12.3)	174 (88.8)	5 (2.6)	17 (8.7)
Ethambutol^b^	189 (13.4)	143 (75.7)	13 (6.9)	21 (11.1)	6.4 (2.5–13.1)	164 (86.8)	7 (3.7)	17 (9.0)
*MDR-TB drugs*								
Bedaquiline	33 (2.3)	9 (27.3)	10 (30.3)	NA	10.2 (4.6–15.9)	27 (81.8)	3 (9.1)	3 (9.1)
Levofloxacin	95 (6.8)	76 (80.0)	17 (17.9)	NA	3.8 (2.0–8.9)	90 (94.7)	3 (3.2)	2 (2.1)
Moxifloxacin	149 (10.6)	87 (58.4)	56 (37.6)	NA	7.2 (3.3–13.2)	135 (90.6)	7 (4.7)	7 (4.7)
Linezolid	155 (11.0)	110 (71.0)	41 (26.5)	NA	5.4 (1.9–10.6)	130 (83.9)	8 (5.2)	17 (11.0)
Clofazimine	28 (2.0)	6 (21.4)	9 (32.1)	NA	9.4 (5.2–17.3)	24 (85.7)	2 (7.1)	2 (7.1)
Total	1407 (100)	960 (68.2)	337 (24.0)	23 (1.6)	7.3 (3.3–15.1)	1171 (83.2)	105 (7.5)	130 (9.2)

NA, not applicable.

^a^The analytical method was not reported for 87 measurements (6.2%).

^b^One result for ethambutol could not be assessed. The weighed-in concentration was below the lower limit of quantification of the analytical method of the laboratory in question and consequently no quantitative result was reported.

**Figure 1.**
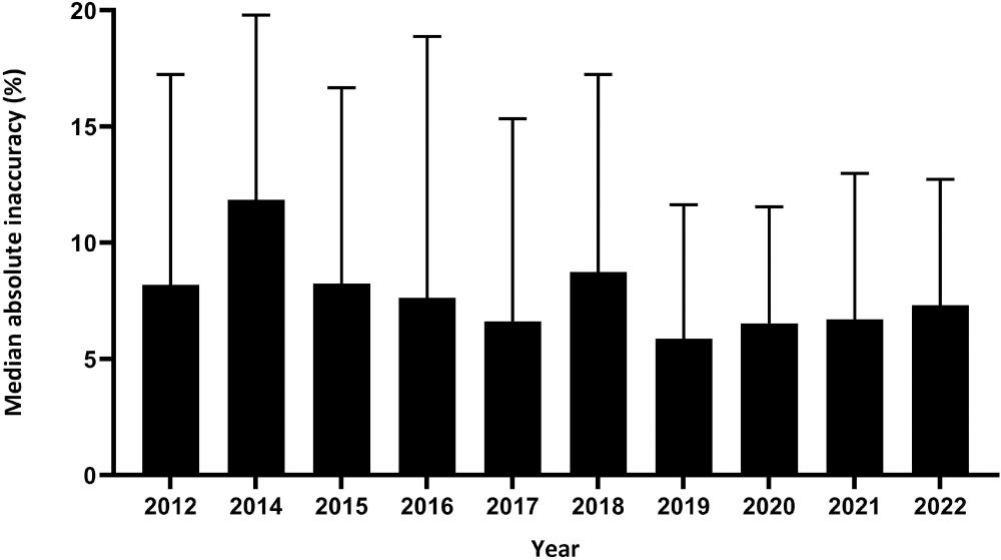
Median absolute inaccuracies (+ upper quartile limit) per year of the QC programme. No rounds were organized in 2013.

Overall, 1171 measurements (83.2%) were accurate according to the predefined range of 80%–120%, whereas 105 measurements (7.5%) were below 80% and 130 measurements (9.2%) were above 120%. The median absolute inaccuracy was 7.3% (IQR, 3.3%–15.1%). The median absolute inaccuracies per year of the programme are depicted in Figure 1.

### Results per drug or metabolite

Laboratories produced the highest percentage of accurate measurements for levofloxacin, moxifloxacin and acetyl-isoniazid (all above 90%) (Table 1). Isoniazid, rifampicin and desacetyl-rifampicin had the lowest percentages of accurate measurements (all below 80%), whereas 80%–90% of measurements of the other drugs and metabolites yielded accurate results. In accordance with this, the order of compounds based on median absolute inaccuracies, from low to high, was: levofloxacin, linezolid, acetyl-isoniazid, desacetyl-rifabutin, ethambutol, pyrazinamide, moxifloxacin, rifabutin, clofazimine, bedaquiline, rifampicin, isoniazid and desacetyl-rifampicin (Table 1).

### Results per concentration level

**Figure 2.**
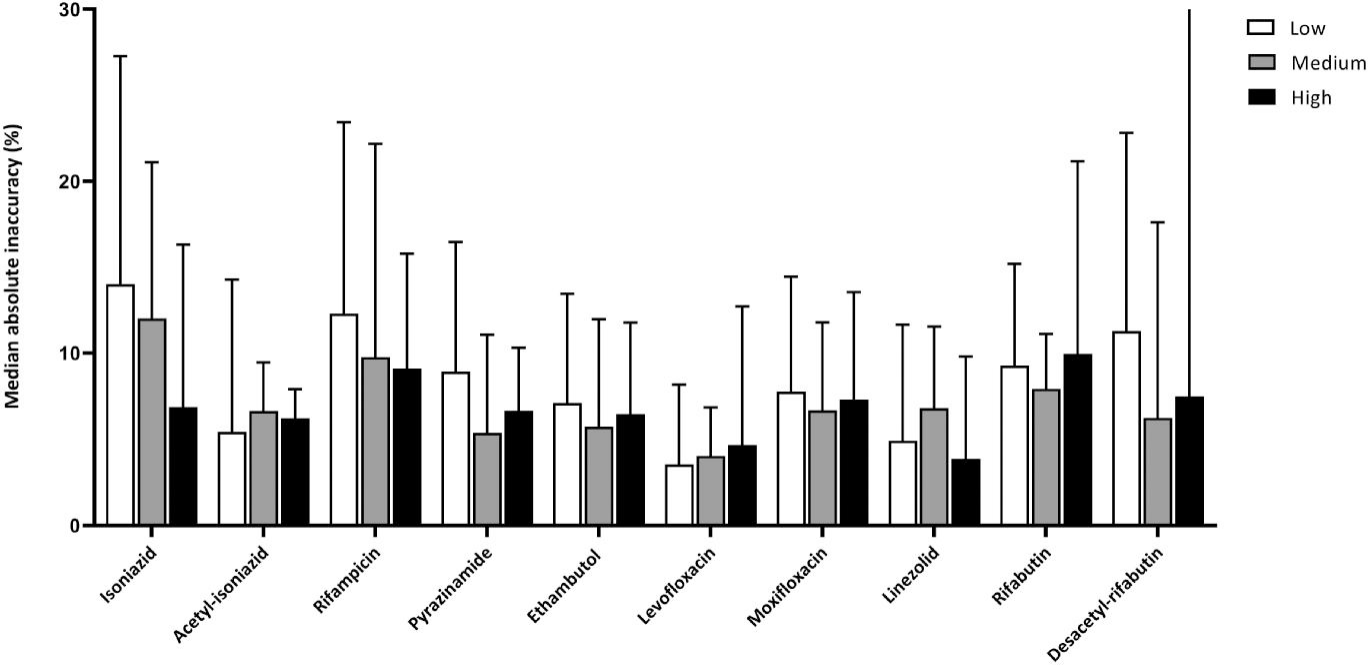
Median absolute inaccuracies (+ upper quartile limit) per drug and concentration level (low, medium, high). For desacetyl-rifabutin, the upper quartile limit for the high concentration level (56.0%) is not captured in the figure.

The percentages of measurements with sufficient accuracy were 81.5%, 84.0% and 85.0% for the low, medium and high concentrations, respectively. Median absolute inaccuracies were 8.1% (IQR, 3.5%–16.2%), 6.9 (IQR, 3.2%–14.6%) and 6.7% (IQR, 3.2%–13.0%) for the low, medium and high concentrations, which means that results were comparable for the different concentration levels. The median absolute inaccuracies for each drug and concentration level are depicted in Figure 2. Bedaquiline, clofazimine and desacetyl-rifampicin were excluded from this analysis because less than nine (six, six and four, respectively) weighed-in concentrations were available.

### Results per analytical technique

The percentages of accurate measurements with LC-MS, HPLC and GC-MS were 84.8%, 78.0% and 100%, respectively. Median absolute inaccuracies were: 6.9% (IQR, 3.2%–14.2%) for LC-MS, 8.8% (IQR, 3.7%–17.6%) for HPLC and 3.9% (IQR, 1.9%–8.1%) for GC-MS.

### Results per performing laboratory

There were large differences in the number of measurements per laboratory (median, 27; range, 2–180). The median percentage of accurate measurements per laboratory was 85.7% with a range of 0% to 100%. The laboratories with 0% (*n* = 1) or 100% (*n* = 3) accurate measurements only reported a small number of measurements (2–6). The median absolute inaccuracies ranged from 3.6% to 27% for the 31 participating laboratories.

### Mixed model linear regression analysis

Eight outliers, with absolute inaccuracies of more than 200% (range 225%–82 567%), were excluded from this analysis because they could substantially affect the mean absolute inaccuracy. This cut-off was selected subjectively. Although the source of these inaccuracies was unknown, such large deviations were assumed to be the result of human errors rather than analytical errors. In agreement with the descriptive findings, there were significant differences in mean absolute inaccuracy between the anti-TB drugs (*P* < 0.001). No significant differences in absolute inaccuracy were found between different concentration levels and analytical techniques (*P* = 0.54 and *P* = 0.26, respectively). The percentage of residual variance in the absolute inaccuracy, attributable to the performing laboratory, was 14.3%.

### Clinical cases

A clinical case was included in the majority of the rounds. An overview of the topics is depicted in Table 2. These topics reflected typical indications for TDM of anti-TB drugs and highlighted specific drug characteristics relevant to each of the clinical scenarios, the limitations to TDM for anti-TB drugs, and specific items relevant to TDM. One case is depicted as an illustrative example (see Text S1).

**Table 2.** Overview of cases during 10 years of the programme

Year plus round	Clinical scenario
2012	*No case*
2014 first case	Drug-susceptible TB: TB/HIV coinfection and slow response
2014 second case	MDR-TB: pyrazinamide and moxifloxacin, TDM of amikacin
2015.1	Drug-susceptible TB: diabetes mellitus and renal insufficiency
2015.2	Drug-susceptible TB: TB meningitis
2016.1	MDR-TB: moxifloxacin and linezolid
2016.2	Drug-susceptible TB: renal insufficiency
2017.1	Drug-susceptible TB: drug-induced liver injury
2017.2	Drug-susceptible TB: abdominal TB and malabsorption
2018.1	Drug-susceptible TB: TB meningitis
2018.2	MDR-TB: levofloxacin and linezolid
2019.1	Drug-susceptible TB: diabetes mellitus and QTc interval prolongation
2019.2	Drug-susceptible TB: osteo-articular TB
2020.1	Drug-susceptible TB: TB/HIV coinfection and drug interactions with rifabutin
2020.2	MDR-TB, moxifloxacin and linezolid and the relevance of MIC values
2021.1	Drug-susceptible TB: relapse TB and slow response
2021.2	*No case*
2022.1	Drug-susceptible TB: drug-induced liver injury and renal insufficiency
2022.2	*No case*

## Discussion

The number of participants in the international, external QC programme for the measurement of anti-TB drugs has increased from 7 laboratories in 2012 to a total of 31 laboratories that had participated (in at least one round) by 2022.^7^ The programme covers in total 13 different anti-TB drugs and metabolites. This expansion of the programme and the overall results of 10 years of the programme illustrate the continuous need for a programme that provides an external assessment of the performance of laboratories.

The majority of drug concentrations that were reported in the programme (58%) related to the first-line anti-TB drugs isoniazid, rifampicin, pyrazinamide and ethambutol. This is not surprising, considering that most new TB cases worldwide (96.1% in 2022) succumb to drug-susceptible TB.^1^ The provision of external QC for measurement of the pivotal second-line anti-TB drugs bedaquiline, linezolid, the fluoroquinolones moxifloxacin and levofloxacin, and clofazimine is, however, equally important, because MDR-TB is a growing public health problem and its treatment warrants further optimization and individualization.^1^ The programme will likely continue to extend with other MDR-TB drugs.

Most analyses were performed with LC-MS, which reflects the increasing use of this technique in pharmacokinetic laboratories. This is probably related to the advantages of this technique, including the relative ease to develop analytical methods for simultaneous measurement of multiple compounds with different chemical structures, limited sample preparation, limited interference by endogenous compounds and other drugs, the achievement of lower limits of quantitation, and shorter run times, as compared with HPLC with other modes of detection.^11^

The actual 10 year results of this international QC programme for anti-TB drugs show that 83.2% of all measurements were sufficiently accurate (within 80%–120% of weighed-in concentrations). This is comparable with results from other QC programmes, with 81.0%, 80.8% and 83.6% accurate measurements for programmes focusing on antimicrobial, antifungal and antiretroviral drugs, respectively.^12–14^ Furthermore, the percentage of accurate measurements in the first round of our programme was 82.7% and thus nearly identical to our 10 year results.^7^ However, because both the participating laboratories and the included drugs have differed over the years, comparisons regarding analytical performance should be made with caution. Regardless, one in six (16.8%) measurements is inaccurate, which, although comparable with other programmes, is clearly suboptimal. Accurate measurements are essential to provide reliable results in pharmacokinetic studies that are meant to substantiate existing or new dosing regimens. In patient care, inaccurate measurements may lead to inadequate dose adjustments when applying TDM.

Our descriptive analysis and mixed model linear regression revealed that the absolute inaccuracy was related to the measured anti-TB drug and to the performing laboratory, but not to the concentration level of the drug or to the analytical technique used. We have questioned whether infrequently analysed drugs were only measured by more experienced laboratories, possibly influencing the absolute inaccuracies of these compounds. However, follow-up exploratory analysis did not reveal such an association (data not shown). Isoniazid and rifampicin had relatively high median absolute inaccuracies (10.9% and 10.2%, respectively). Isoniazid is known to be relatively unstable at room temperature. Bioanalysis of rifampicin may require an adjuvant (ascorbic acid) to prevent degradation, as evidenced by many authors.^15–18^ Adequate storage conditions (e.g. −80°C) and timely processing of plasma samples are recommended.^15^ As to the performing laboratory, large variability between laboratories was observed in terms of percentage of accurate measurements and median absolute inaccuracies. This between-laboratory variability probably relates to differences in intralaboratory (internal) QC, which includes the rigour of analytical method validation, validation of equipment, training and qualification of technicians, and other intralaboratory QC procedures.

Our QC programme has several strengths. First, it serves participating laboratories across the world and the portfolio of compounds has evolved to include many important anti-TB drugs. To our knowledge, there is only one other international QC programme for the measurement of anti-TB drugs, which so far includes only rifampicin, and no results have yet been published. Secondly, by using human plasma that is no longer freeze-dried (requiring an additional dissolution step), the programme aims to mimic clinical (or research) practice. One exception was made for isoniazid, for which QC samples were prepared in water and subsequently needed to be mixed with blank plasma by participants. This was to ensure no degradation would occur during preparation, shipment or handling of the samples. As a result of the similarities between QC samples and real samples, it can be inferred that the results of the QC programme provide a measure of the intralaboratory quality assurance in the participating laboratories. Still, it cannot be excluded that laboratories made additional efforts to achieve their results in this external QC programme. This means that the results of the programme could also reflect the best performance of the participants. Thirdly, the clinical cases served a unique educational purpose in this and several other programmes of the KKGT section of SKML. Not only did these cases illustrate situations when TDM could be useful in patient care, but also they allowed participants to practise with the application of TDM.

A limitation of our QC programme was that it did not include error evaluation questionnaires like certain other programmes do.^13,19^ Including such forms would allow better assessment of the possible sources of measurement inaccuracies. This will be considered for future rounds.

To summarize, the 10 year results of our external QC programme illustrate the need for continuous external QC for the measurement of anti-TB drugs. Participation of laboratories in the programme either confirms their level of intralaboratory quality assurance or alerts them to inaccuracies and underlying undetected problems, inciting them to optimize their methods or QC procedures. We continue to extend an open invitation to laboratories around the world to participate in this QC programme. Participation in the programme is possible by subscribing through www.skml.nl.

## Supplementary Material

dkae105_Supplementary_Data

## Supplementary data

Figure S1 and Text S1 are available as Supplementary data at *JAC* Online.
