# Supplementary material for: Ten-year results of an international external quality control programme for measurement of anti-tuberculosis drug concentrations
Source: J Antimicrob Chemother. 2024 Apr 6;79(6):1346–52. doi: 10.1093/jac/dkae105 (PMC11144482; doi:10.1093/jac/dkae105)
Supplement: dkae105_Supplementary_Data [file dkae105_supplementary_data.docx]

**Supplementary data**


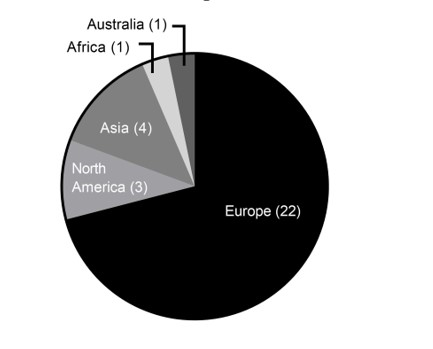
 **Figure S1.** Global distribution of participating laboratories

**Text S1**

**Example of a clinical case**

The pharmacological laboratory was called by a concerned medical doctor from a municipal health center who treated a 24 year old male patient of 48 kg with drug-susceptible pulmonary TB and large pulmonary cavities. The patient had presented with relapse TB with a mycobacterium that was still susceptible to all first-line TB drugs. Unfortunately, the patient did not show sputum culture conversion after 3 months of his second course of directly observed treatment, he did not gain weight at all, kept on coughing and remained sick. The municipal health center had never considered TDM ‘as it was not supported by randomized trials’ and ‘it was considered to be an expensive examination’. In case of this severely ill patient, the medical doctor was still advised to perform TDM. Samples were obtained at two and 6 hours after the dose to ‘catch’ peak plasma concentrations of isoniazid, rifampicin and pyrazinamide (ethambutol had been stopped). Peak concentrations were as follows: rifampicin: 5.1 mg/L, isoniazid: 2.0 mg/L, acetyl-isoniazid: 3.9 mg/L and pyrazinamide: 39.4 mg/L.

Your advice based on these results would be:

A: Keep the drug doses as they are

B: Increase the dose of isoniazid, monitor transaminases

C: Increase the dose of isoniazid and rifampicin, monitor transaminases

D: Increase the dose of isoniazid, rifampicin and pyrazinamide, monitor transaminases

**Evaluation of the case**

This is an example of a patient with relapse TB who does not show an adequate response to TB drugs. Based on this suboptimal response, the treatment regimen was empirically extended - the patient is still using pyrazinamide after 3 months of treatment.

There is an indication for Therapeutic Drug Monitoring (TDM) as both the relapse TB and the current suboptimal response may be due to low drug concentrations [1]. Indeed, TDM proved to be of help in detecting low TB drug concentrations as a possible cause of the slow treatment response in this patient. The measured peak concentration of isoniazid (2.0 mg/L) is below the normal (reference) range for a once daily 300 mg (5 mg/kg) dose (3-6 mg/L [2]). This may be explained by the patient being a fast acetylator. The acetyl-isoniazid to isoniazid ratio is 2.0. At a sampling time of 3 h after the dose, a ratio above 1.5 is an indication for a fast acetylator [3]. The peak concentration of rifampicin (5.1 mg/L) is also below the reference range of 8-24 mg/L [2]. For this reason, it seems wise to increase the dose of isoniazid and rifampicin.
The pyrazinamide peak concentration (39.4 mg/L) is within the normal range (20-60 mg/L [2]) and also above a cut-off value of 35 mg/L that is used by some [4]. It should be realized, however, that studies in the hollow fiber model and in animals as well as some clinical data (e.g. [5]) suggest that higher pyrazinamide exposures are more efficacious. In this case of non-response to TB drugs and a sick patient, an increase in the dose of pyrazinamide may be justified under monitoring of transaminases.

TDM was considered ‘expensive’ by the doctor, but it might prevent prolonged treatment in many similar patients and is cost-effective as shown by ‘back of the envelope’ calculations. Indeed randomized trials have not shown the value of TDM for TB drugs and many other drugs, but in clinical practice it has shown added value in selected cases.

**References**

1. Magis-Escurra C, van den Boogaard J, Ijdema D, Boeree M, Aarnoutse R. Therapeutic drug monitoring in the treatment of tuberculosis patients. Pulm Pharmacol Ther 2012;25(1):83-6.

2. Alsultan A, Peloquin CA. Therapeutic drug monitoring in the treatment of tuberculosis: an update. Drugs. 2014;74(8):839-54

3. Hutchings A, Routledge PA. A simple method for determining acetylator phenotype using isoniazid. Br J Clin Pharmacol 1986;22:343– 345.

4. Chideya S, Winston CA, Peloquin CA, et al. Isoniazid, rifampin, ethambutol, and pyrazinamide pharmacokinetics and treatment outcomes among a predominantly HIV-infected cohort of adults with tuberculosis from Botswana. Clin Infect Dis 2009;48:1685–94.

5. Pasipanodya JG, McIlleron H, Burger A, Wash PA, Smith P, Gumbo T. Serum drug concentrations predictive of pulmonary tuberculosis outcomes. J Infect Dis 2013;208(9):1464-73.
